# Temporal Dynamics of Inhalation-Linked Activity across Defined Subpopulations of Mouse Olfactory Bulb Neurons Imaged *In Vivo*

**DOI:** 10.1523/ENEURO.0189-19.2019

**Published:** 2019-06-27

**Authors:** Shaina M. Short, Matt Wachowiak

**Affiliations:** Department of Neurobiology and Anatomy, University of Utah, Salt Lake City, UT 84112

**Keywords:** olfaction, inhalation, glomerulus, olfactory sensory neuron, interneuron, tufted cell, mitral cell, circuit, calcium imaging

## Abstract

In mammalian olfaction, inhalation drives the temporal patterning of neural activity that underlies early olfactory processing. It remains poorly understood how the neural circuits that process incoming olfactory information are engaged in the context of inhalation-linked dynamics. Here, we used artificial inhalation and two-photon calcium imaging to compare the dynamics of activity evoked by odorant inhalation across major cell types of the mouse olfactory bulb (OB). We expressed GCaMP6f or jRGECO1a in mitral and tufted cell (MTC) subpopulations, olfactory sensory neurons (OSNs), and two major juxtaglomerular interneuron classes and imaged responses to a single inhalation of odorant. Activity in all cell types was strongly linked to inhalation, and all cell types showed some variance in the latency, rise times, and durations of their inhalation-linked response. Juxtaglomerular interneuron dynamics closely matched that of sensory inputs, while MTCs showed the highest diversity in responses, with a range of latencies and durations that could not be accounted for by heterogeneity in sensory input dynamics. Diversity was apparent even among “sister” tufted cells innervating the same glomerulus. Surprisingly, inhalation-linked responses of MTCs were highly overlapping and could not be distinguished on the basis of their inhalation-linked dynamics, with the exception of a subpopulation of superficial tufted cells expressing cholecystokinin (CCK). Our results are consistent with a model in which diversity in inhalation-linked patterning of OB output arises first at the level of sensory input and is enhanced by feedforward inhibition from juxtaglomerular interneurons which differentially impact different subpopulations of OB output neurons.

## Significance Statement

Inhalation drives the temporal patterning of neural activity that underlies olfactory processing and rapid odor perception, yet the dynamics of the neural circuit elements mediating this processing are poorly understood. By comparing inhalation-linked dynamics of major olfactory bulb (OB) subpopulations, we find that diversity in the timing of neural activation arises at the level of sensory input, which is then mirrored by inhibitory interneurons in the glomerular layer. Temporal diversity is higher among OB output neurons, with different subpopulations showing distinct but nonetheless highly overlapping ranges of inhalation-linked dynamics. These results implicate feedforward inhibition by glomerular-layer interneurons in diversifying temporal responses among output neurons, which may be important for generating and shaping timing-based odor representations during natural odor sampling.

## Introduction

Temporal patterning of neural activity is a fundamental aspect of information coding and processing by neural circuits. In the mammalian olfactory system, the primary driver of temporally patterned activity is inhalation of air through the nasal cavity. Inhalation delivers transient pulses of odorant to the olfactory epithelium, and so determines the initial temporal structure of olfactory sensory input to the brain and drives the temporal patterning of activity at subsequent processing stages (Macrides and Chorover, 1972; Onoda and Mori, 1980; Sobel and Tank, 1993; Kepecs et al., 2006; Schaefer et al., 2006; Schaefer and Margrie, 2007; Wachowiak, 2011). Behavioral and psychophysical studies have shown that odor percepts are formed within the time of a single inhalation (150–250 ms for rodents, ∼400 ms for humans; Laing, 1986; Johnson et al., 2003; Kepecs et al., 2007), and neurophysiological studies have demonstrated that the temporal pattern of neural activity elicited by a single inhalation of odorant can robustly encode odorant identity and intensity (Uchida and Mainen, 2003; Kepecs et al., 2007; Wesson et al., 2008; Wesson et al., 2009; Cury and Uchida, 2010; Shusterman et al., 2011; Rebello et al., 2014). Thus, understanding how inhalation-linked temporal patterns of activity are generated and shaped by neural circuits in the early olfactory pathway is fundamental to understanding olfactory information processing.

Neural circuits in the olfactory bulb (OB) mediate the first steps in processing olfactory inputs: here, olfactory sensory neurons (OSNs) drive excitation of mitral and tufted cells (MTCs), the principal output neurons of the OB, as well as activate multiple inhibitory circuits within and between OB glomeruli (Wachowiak and Shipley, 2006). This juxtaglomerular inhibition is hypothesized to play a critical role in shaping MTC responses to odorants (Gire and Schoppa, 2009; Shao et al., 2009, 2012; Fukunaga et al., 2014; Banerjee et al., 2015; Liu et al., 2016b) yet the temporal dynamics of activity among different juxtaglomerular interneurons with respect to inhalation have not been well-characterized. Two types of juxtaglomerular interneuron classes, periglomerular (PG) and short axon (SA) cells, are hypothesized to mediate feedforward and lateral inhibition, respectively, with differential impacts on the temporal dynamics of MTC responses (Aungst et al., 2003; Shao et al., 2009, 2012; Shirley et al., 2010; Fukunaga et al., 2012; Liu et al., 2013, 2016b; Whitesell et al., 2013; Banerjee et al., 2015; Najac et al., 2015; Geramita and Urban, 2017). Additionally, feedback inhibition mediated by reciprocal connections between MTCs to PG or SA cells may also shape MTC temporal patterning (Najac et al., 2015).

Here, we sought to better characterize the temporal dynamics of inhalation-driven activity among major circuit elements in the OB to refine models of OB circuit function during naturalistic odorant sampling *in vivo*. We used cell type-specific imaging with GCaMP-based reporters to record from different subpopulations of MTCs as well as presumptive PG cells, SA cells, and OSN inputs. We used an artificial inhalation paradigm to examine responses to a single inhalation of odorant with high fidelity and to precisely compare inhalation-linked response patterns across experiments and between cell types. We found distinct differences in the inhalation-linked temporal patterns of activity among different cell types, with PG and SA cell populations showing faster responses to inhalation than either mitral or tufted cells, and a range of excitatory MTC response dynamics that could not be accounted for by diversity in the dynamics of OSN inputs. At the same time, we observed a great deal of overlap in the inhalation-linked temporal patterns of activity among superficial tufted and mitral cells. Overall, these results support circuit models in which juxtaglomerular interneurons mediate rapid feedforward inhibition that contributes to diverse inhalation-linked temporal patterning in both MTCs.

## Materials and Methods

### Animals

Genetically-engineered mice expressing Cre recombinase (Cre) targeted to specific neuronal populations were used for experiments. Mice were either crossed to the Ai95 GCaMP6f reporter line (The Jackson Laboratory (JAX), stock #024105) or injected with a viral vector. The mouse strains using included GAD2-IRES-Cre mice (JAX, stock #010802), TH-Cre (JAX, stock #008601), DAT-IRES-Cre mice (JAX, stock #006660), OMP-Cre (JAX, stock #006668), PCdh21-Cre (Gensat, stock #030952-UCD; Nagai et al., 2005), Tbet-Cre (JAX, stock #024507), Thy1-GCaMP6f transgenic mice (JAX, stock #024339, line GP5.11) mice, and CCK-IRES-CRE (JAX, stock #012706). Mice were on average 4.6 months of age by completion of data collection. Both female (46) and male (54) mice were used. Mice were housed up to five per cage in a 12/12 h light/dark cycle. Food and water were provided ad libitum. Each procedure was performed following the National Institutes of Health Guide for the Care and Use of Laboratory Animals and approved by the University of Utah institutional animal care and use committee.

### Viral vector expression

Cre-dependent expression of GCaMP6f (AAV2/9, AAV2/1, or AAV2/5 stereotypes of hSyn.Flex.GCaMP6f) or jRGECO1a (AAV2/9 or AAV2/5 hSyn.Flex.NES.jRGECO1a.WPRE.SV40) was achieved with the injection of viral vectors. All viruses were obtained from the University of Pennsylvania Viral Vector Core. Injections were made into the dorsal OB under isoflurane anesthesia (0.5–2% in O_2_). Where applicable, mice used for virus injection were homozygous for the allele driving Cre expression. Using a stereotaxic head holder and drill, a small craniotomy (0.5–1 mm) was made on the dorsal surface of the OB. A glass pipette was lowered to a depth 50–150 μm to target PG or SA interneurons or 200–400 μm to target MTC populations. Mice were single housed following the surgery and imaged 14–28 d after injection.

### *In vivo* imaging

Two-photon imaging was performed in acutely anesthetized mice. Initially, pentobarbital (50 mg/kg) was used during the implantation of a double tracheotomy (Wachowiak and Cohen, 2001; Bozza et al., 2004; Spors et al., 2006), after which isoflurane (0.5–2% in O_2_) was delivered directly to the tracheotomy tube, bypassing the nose. By controlling negative pressure through the sniff tube, each inhalation lasted exactly 150 ms and had a flow rate of 150 ml/min (300 ml/min for two nostrils). The onset of each inhalation was set to occur every 4 s (0.25 Hz), which enabled individual inhalation evoked calcium transients to be averaged. Next, a custom head bar was implanted, a craniotomy was made, and a coverslip was implanted using 2.5% low-melting-point agarose over the dorsal OB for imaging (Wachowiak et al., 2013). Throughout surgeries and while imaging, the body temperature was maintained at 37°C with a heating pad and the heart rate at ∼400 beats per minute.

Imaging data were collected using either a Moveable Optical Microscope (Sutter Instruments) coupled to a Mai Tai HP pulsed Ti:Sapphire laser (Newport Corp.) and controlled by Scanimage 5.1 (Pologruto et al., 2003), or a Neurolabware microscope coupled to a Cameleon UltraII laser (Coherent) and controlled by Scanbox software. Both setups used resonance-based scanning and GaAsP photomultipliers (Hamamatsu H10770B) for light collection, and images were collected at a frame rate of 15.5 Hz. A 16 × 0.8 N.A. (Nikon) objective was used in all experiments. For dual-color imaging, a Fidelity 1070 nm femtosecond laser was used simultaneous with 920-nm illumination and emission filters were used to separate green (520/65 nm) and red (641/75 nm) emission (Sun et al., 2017). The average power delivered to the sample during imaging was 20–60 mW.

### Analysis of imaging data

**Figure 1.**
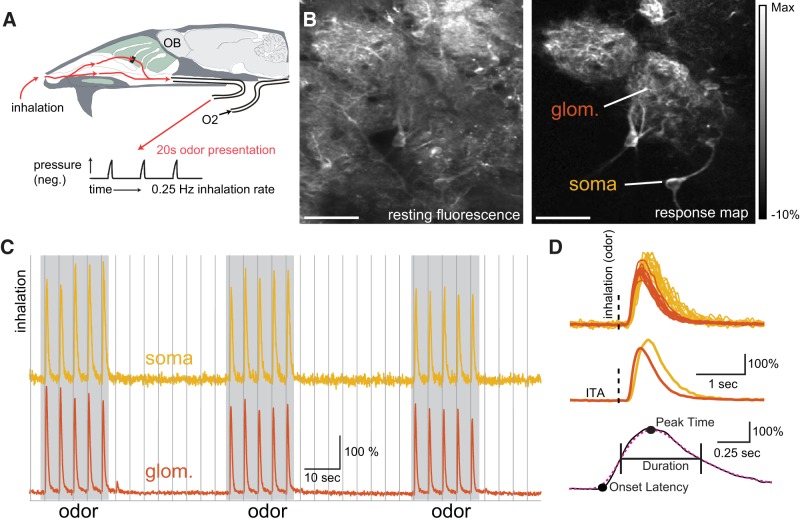
Imaging inhalation triggered temporal dynamics of OB neuron subtypes. ***A***, Schematic of preparation for artificial inhalation in the anesthetized mouse. Inhalations were generated by a vacuum pulse applied to nasopharynx at 0.25 Hz. ***B***, Example resting fluorescence (left) and inhalation triggered average (ITA) fluoresce change map taken from a glomerulus (glom.) and interneuron somata (soma) in the glomerular layer in a GAD2-Cre:GCaMP6f reporter cross during odorant stimulation. White scale bar = 50 μm. Presumptive PG cell somata and glomerular boundaries are difficult to distinguish in the resting fluorescence image, but are clearly apparent in the response map. ***C***, Traces showing the fluorescence signal imaged from the glomerular neuropil and soma of an associated GAD2+ neuron, from the example in ***B***. Traces show a typical imaging epoch, with three 20-s odor stimuli (gray) presented during 0.25-Hz inhalation (vertical lines). ***D***, Overlaid inhalation triggered calcium signals (top) and corresponding ITAs (middle) taken from the glomerulus and soma during each inhalation in the presence of odorant. Vertical dashed line indicates inhalation onset. Bottom, Schematic illustrating definition of onset latency, time to peak, and response duration. Onset latency was calculated from the ITA trace (solid black line) when the response initially surpassed at least four consecutive frames, 4 SD above baseline. Time to peak (time to maximum response) and response duration (time between the half max and half min points) were calculated from the ITA after applying the Gaussian weighted box filter (dotted magenta line).

Maps of inhalation-triggered fluorescence changes (i.e., ITA response ΔF maps) were generated by choosing 15 frames before and after odorant inhalation. Imaging trials consisted of three periods of odorant presentation, each lasting 20 s (i.e., five inhalations at 0.25 Hz), with a 32-s interval between presentations. Responses to inhalation of clean air were taken from the last 10 inhalations from the interstimulus period to ensure that all odorant had cleared from the nose following the stimulus presentation. Note, small transients that immediately followed odor stimulation (Fig. 1*C*, 7th inhalation) likely reflect incomplete clearance of odorants from in front of, or inside, the nasal cavity. Responses to inhalation of clean air needed to be consistent across interstimulus periods of subsequent trials of odorant or a blank (medium-chain triglycerides) no-odor control presentations for the cell to be counted as responding to inhalation alone. For display, ITA response maps were smoothed with a Gaussian filter with sigma 1.25 pixels. Regions of interest (ROIs) were selected manually from ITA response maps or from resting fluorescence images. Fluorescence time series were extracted by averaging all pixels in a ROI using custom MATLAB scripts. All time series data were sampled to 150 Hz using the MATLAB piecewise cubic interpolation functions interp1 and pchip. In all cases, ΔF/F was calculated as (ΔF/F = (F – F_o_)/F_o_), with F_o_ being the mean fluorescence before the inhalation, averaged for each inhalation. However, for population level analyses, signals were averaged across three trials of 20-s odorant presentations (Fig. 1). Excitatory events were defined as ITA responses that were >4 SD above the ITA baseline signal, which was defined as 1 s before inhalation, whereas inhibitory events were defined as ITA responses reaching >3 SD below baseline.

### Analysis of temporal dynamics

**Figure 2.**
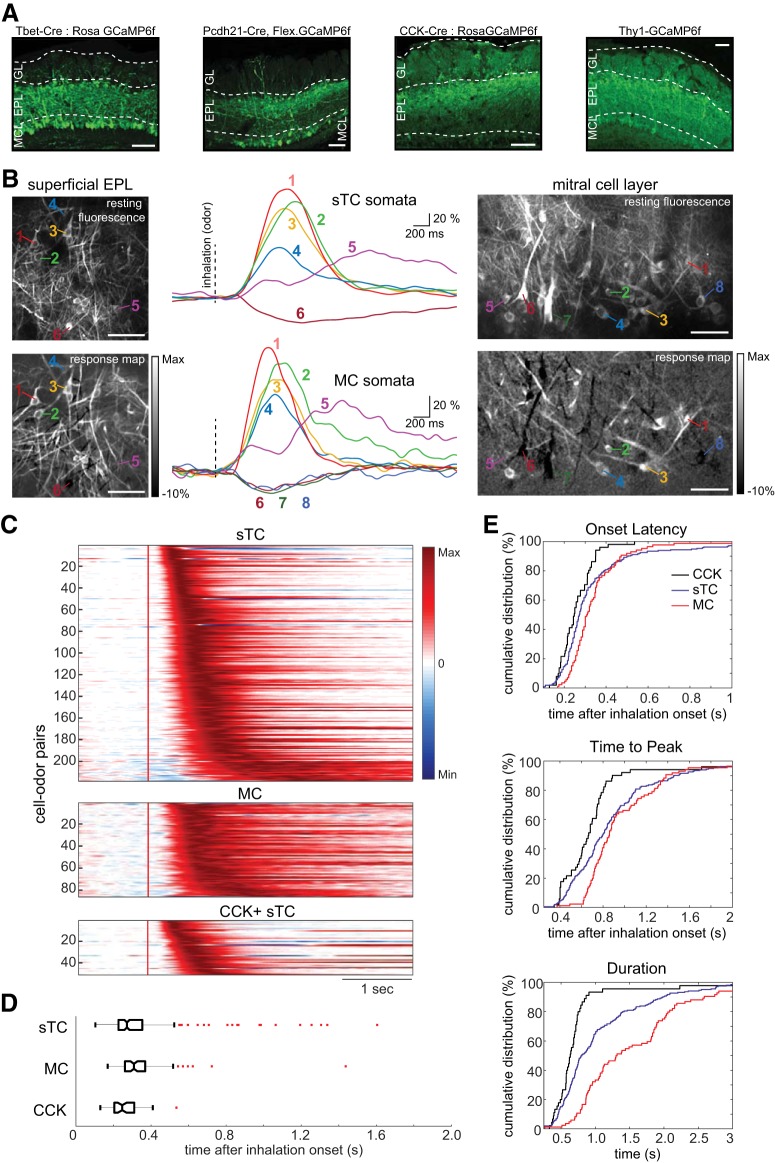
Inhalation-linked dynamics of mitral and superficial tufted cells are diverse and highly overlapping. ***A***, GCaMP6f expression targeted to MC and sTC populations using reporter crosses to target GCaMP6f expression in Tbx21+, CCK+, and Thy1+ MTC populations and virus injections into the OB to target GCaMP6f expression to PCdh21+ MTCs. Glomerular layer (GL), external plexiform layer (EPL), and mitral cell layer (MCL) are labeled using dotted white lines. ***B***, Resting fluorescence (top) and odor-evoked ITA response maps (bottom) of sTCs imaged from superficial EPL (left images) and MCs imaged from the MCL (right images). Middle column shows ITA traces from sTCs (top) and MCs (bottom) indicated in the images. Signals were low-pass filtered at 3 Hz before averaging and scaled relative to the preinhalation baseline (i.e., ΔF/F). EPL and MCL images were taken from fields of view that were immediately above and below each other in the same Tbet-Cre: Rosa-GCaMP6f mouse during stimulation with the same odorant (methyl valerate). All white scale bars = 50 µm. Vertical dashed lines indicate inhalation onset. ***C***, Pseudocolor plots of ITA responses for all excitatory responses in sTC (top), MC (middle), and CCK^+^ sTCs (bottom); each row shows a different cell-odor pair. Each trace (row) was normalized to its own max and negative max amplitudes on a scale from –1 to 1. Red vertical line indicates inhalation onset. ***D***, Box and whisker plots of sTC, MC, and CCK^+^ sTC onset latency distributions. Outliers marked with red dashes. ***E***, Cumulative distribution plots of onset latency (top), time to peak (middle), and duration (bottom) for MC, sTCs, and CCK^+^ sTC ITAs.

Onset latency was calculated as the first time point in which the following four frames of an ITA trace were above the threshold for a significant excitatory response (4 SD above baseline, which was taken from a 1-s prestimulus window). Peak response amplitudes and time to peak values were calculated from ITAs that were filtered using a Gaussian-weighted moving average filter with a window length of 270 ms. From this filtered trace, response duration was calculated as the time from 50% of peak response on the rising slope of the signal to 50% of peak on the decaying slope of the signal. Time to peak was calculated from the maximum value of the filtered trace. For pseudocolor plots of ITA responses (Fig. 2*C*), the mean of the 1-s prestimulus window was subtracted from the response time series and smoothed using a Gaussian filter with a window length of 100 ms; the resulting trace was normalized to its own maximum and negative maximum amplitudes for pseudocolor display.

### Experimental design and statistical tests

**Figure 3.**
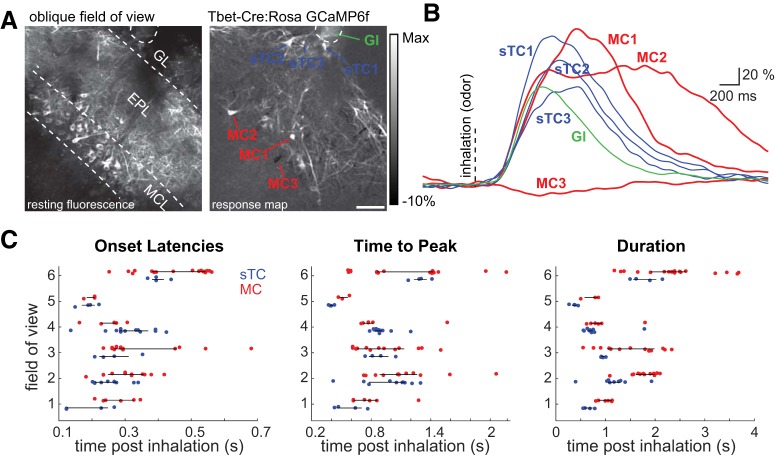
Diversity of inhalation-linked dynamics of MCs and sTCs within the same field of view. ***A***, Resting fluorescence and odor-evoked ITA response map showing sTCs and MCs imaged in the same field of view using an oblique imaging plane in a Tbet-Cre: Rosa-GCaMP6f mouse during stimulation with methyl valerate. Gl, glomerular neuropil. ***B***, Traces showing ITAs from the sTCs and MCs shown in ***A***, illustrating temporal diversity of inhalation-linked responses across different cell types imaged simultaneously. Vertical dashed line indicates inhalation onset. ***C***, Plots comparing latency, peak time, and duration of ITAs from sTCs (blue) and MCs (red) imaged in the same field of view or successive z-planes, allowing for paired comparisons (see text). Black horizontal line spans the interquartile range. White scale bar = 50 μm.

Statistical details of experiments are listed in the Results section. All datasets (onset latency, time to peak, and half width response durations across all superficial tufted cell (sTC), mitral cell (MC), OSN, PG cell, and SA cell populations) rejected the null hypothesis for one-sample Kolmogorov–Smirnov test for normality, therefore non parametric statistical tests were performed as stated throughout the methods. A Wilcoxon signed-rank test tested was used for paired comparisons across two groups (MTC populations; Fig. 3), Mann–Whitney *U* test was used for unpaired comparisons across two groups (MTC population; Fig. 2), and the Kruskal–Wallis test was used for comparisons across all subpopulation (Fig. 6). *Post hoc* multiple comparisons were performed using Tukey’s honest significant difference criterion (Fig. 6). For comparisons of glomerular neuropil dynamics to PG and sTC somatic responses (Fig. 7), an unpaired *t* test was used to statistically compare SD across sTC and PG glomerulus-cell pairs. All statistical tests were performed in MATLAB. Statistical significance was set at *p* < 0.05 for all tests.

### Olfactometry

A custom olfactometer controlled by Labview software was used to present odorants, as previously described (Bozza et al., 2004; Verhagen et al., 2007). Odorant concentration was controlled by diluting from saturated vapor in filtered, ultra-pure air. Odorants were present for 20 s at 1–5% saturated vapor. Each odorant presentation was separated by a 32-s interstimulus interval. Odorants were obtained at 95–99% purity (Sigma-Aldrich) and stored under nitrogen. Some odorants were diluted in mineral oil to achieve final concentrations at the animal’s nose of 1–20 ppm at 1% saturated vapor. Odorants tested included ethyl butyrate, methyl valerate, butyl acetate, 2-hexanone, ethyl tiglate, 2-methyl pentanal, 2-hydroxyacetophenone, and hexyl acetate. Odorants were delivered 0.5–1.0 cm in front of the mouse’s nose. Filtered, ultra-pure air was delivered to the mouse’s nose in between odorant presentations. A fan positioned behind the animal scavenged excess odorant in the room.

### Histology

An overdose of sodium pentobarbital was used to deeply anesthetize mice before perfusion with PBS followed by 4% paraformaldehyde in PBS. Overnight, heads were postfixed in 4% paraformaldehyde in PBS. Next, the brain was extracted and embedded in 5% agarose. Coronal sections (100–200 μm thick) were made with a Vibratome and mounted onto a glass coverslip before imaging with a FluoView FV1000 Olympus confocal microscope at 10×, 20×, and 40× magnification.

## Results

To compare the dynamics of inhalation-driven activity across different OB neuron populations, we used an artificial inhalation paradigm that allowed for precise comparison of temporal response dynamics across experiments, as described previously (Wachowiak et al., 2013; Díaz-Quesada et al., 2018; Fig. 1*A*). We generated single inhalations at 0.25 Hz to enable inhalation-triggered averaging of responses with, in most cases, minimal adaptation from one inhalation to the next. However, the duration and peak flow rate of each inhalation was chosen to be within the range that occurs in awake, freely-breathing mice. We expressed calcium sensors (typically GCaMP6f) in distinct genetically-defined cell types using either viral vectors or genetic expression strategies, and imaged activity from the somata of the targeted cell types or from glomerular neuropil using two-photon laser scanning microscopy (Fig. 1*B*). A typical imaging epoch consisted of three periods of odorant presentation, each lasting 20 s (i.e., five inhalations at 0.25 Hz), with a 32-s interval between presentations (Fig. 1*C*), yielding a total of 15 inhalations in the presence of odorant. We analyzed response dynamics from inhalation-triggered average traces (ITAs) and determined response magnitudes, latencies, and durations, as done previously (Carey et al., 2009; Fig. 1*D*).

### Inhalation-linked dynamics of mitral/tufted cell subpopulations

We first examined inhalation-linked response dynamics in MTCs. Electrophysiological recordings have shown diverse inhalation-linked temporal patterns among MTCs and distinct differences between these subpopulations as defined by soma depth (Fukunaga et al., 2012; Igarashi et al., 2012). To test whether these differences were reflected in inhalation-linked calcium signals, we selectively expressed GCaMP6f in MTCs via several mechanisms: viral injection (AAV.Flex.GCaMP6f) into the OB of mice expressing Cre-recombinase in protocadherin-21 positive (PCdh21-Cre) neurons (Wachowiak et al., 2013); crossing a Cre-dependent GCaMP6f reporter line with mice expressing Cre in Tbx21-positive (Tbet-Cre) neurons (Haddad et al., 2013) or in cholecystokinin-positive (CCK^+^) neurons (Seroogy et al., 1985); and use of a transgenic mouse line (Thy1-GCaMP6f) selective for expression in MTCs (Dana et al., 2014). Patterns of GCaMP6f expression using PCdh21-Cre, Tbet-Cre, or Thy1-GCaMP6f mice were qualitatively similar, as described previously (Wachowiak et al., 2013) with expression in large numbers of MTCs (Fig. 2*A*). To compare MTCs, we distinguished each population by somatic depth (Fig. 2*B*). We restricted our analysis to somata that were clearly in the mitral cell layer (MCs) and superficial tufted cells (sTCs) just below the glomerular layer, excluding deeper or middle tufted cells in the external plexiform layer.

For both MCs and sTCs, the predominant inhalation-linked response pattern was a transient fluorescence increase, presumably corresponding to a brief spike burst after inhalation. There was substantial diversity in the dynamics of this transient, with different cells showing differences in onset latency, time to peak response, and response duration; such diversity was apparent for different cells of the same type (i.e., sTCs or MCs) imaged within the same preparation during the same odorant presentation (Fig. 2*B*). When comparing only excitatory responses, MCs and sTCs showed statistically significant differences in latency, time to peak, and response duration (*p* = 0.0032, 0.0119 and 3.3e−9, respectively, Mann–Whitney *U* test, MC = 86 cell-odor pairs from five mice, sTC = 218 cell-odor pairs from 16 mice), however, their response dynamics were highly overlapping (Fig. 2*C*,*D*). MCs and sTCs differed most substantially in their response durations, with MCs showing significantly longer-duration responses (median, 1309 ms, interquartile range: 892–1994 ms) compared to sTCs (median, 788 ms, interquartile range: 611–1296 ms; Fig. 2*E*). Another difference observed between sTCs and MCs was their responsiveness to inhalation of clean air: 9.5% of sTCs (22/231 cells tested with clean air) and zero MCs (0/89 cells tested) showed a significant response to inhalation of clean air.

In several cases, we were able to more directly compare sTC and MC response dynamics, either by imaging sTCs and MCs in the same field of view using an oblique imaging plane (*n* = 3 fields of view from two mice; Fig. 3*A*) or by imaging sTC and MC responses to the same odorant in successive trials by shifting the focal plane from the superficial external plexiform layer to the MCs (*n* = 3 paired imaging planes from two mice). Even with this within-preparation comparison, sTC and MC response dynamics were still highly overlapping (Fig. 3*B*). There was no significant difference in the median ITA onset latency when comparing MC and sTC populations within the same preparation or field of view (Wilcoxon signed-rank test, *p* = 0.16, *n* = 6 paired comparisons, 58 total MCs, 38 total sTCs; Fig. 3*C*, left panel). Likewise, there was no significant difference in median sTC and MC ITA time to peak (Wilcoxon signed-rank test, *p* = 0.22; Fig. 3*C*, middle panel). However, the median half width durations of sTC responses were significantly shorter compared to MC responses (Wilcoxon signed-rank test, *p* = 0.03; Fig. 3*C*, right panel). This analysis supports the conclusion that, as a population, sTCs show slightly shorter-duration responses than MCs, but that individual sTCs and MCs overlap substantially in their inhalation-linked response dynamics.

Finally, we measured inhalation-linked responses in sTCs defined by their expression of the peptide transmitter CCK, which likely constitute a subset of Tbx21+, Thy1+, or PCdh21+ sTCs (Seroogy et al., 1985; Liu and Shipley, 1994; Tobin et al., 2010) and have previously shown to exhibit shorter-onset and simpler odorant-evoked responses than MCs (Economo et al., 2016). Consistent with these earlier reports, sTCs imaged from GCaMP6f:CCK-Cre mice (*n* = 52 cell-odor pairs in 3 mice) indeed showed onset latencies that were, as a population, significantly shorter than those of MCs (*p* = 3.7e–5) or the general sTC population (*p* = 0.0333) defined by Tbx21, PCdh21, or Thy1 expression (median onset latency: 247 ms, interquartile range: 206–311 ms; Mann–Whitney test; Fig. 2*C–E*). Over a quarter of the slowest sTCs onset latencies were slower than 90% of that of the CCK^+^ sTC population (Fig. 2*E*). Likewise, CCK^+^ sTCs showed an earlier time to peak than the general sTC population (*p* = 0.0022, Mann–Whitney test). CCK^+^ sTCs showed the largest difference in their response durations, which were uniformly short (median, 635 ms, range: 520–735 ms) and significantly shorter than the general sTC population (*p* = 9.1e−5, Mann–Whitney test). Thus, CCK^+^ sTCs appear to constitute a distinct subpopulation of sTCs with more rapid inhalation-triggered response patterns.

**Figure 4.**
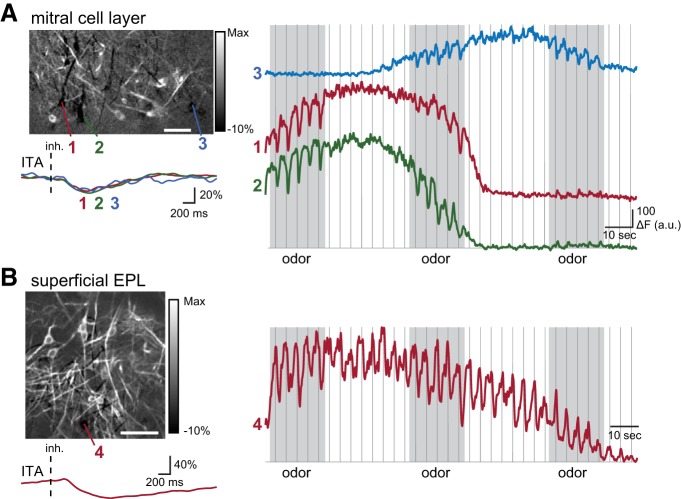
Inhalation-linked suppression in MCs and sTCs. ***A***, left, Odorant-evoked ITA response map including several MCs (Tbet-Cre: Rosa-GCaMP6f mouse) that show suppression of ongoing activity after each inhalation of odorant (methyl valerate, same preparation shown in Fig. 2*B*), with ITA traces from each cell shown below. A 3-Hz low-pass filter was applied to all traces before averaging. Right, Continuous traces from each MC showing fluorescence decrease after odorant inhalation, which is only apparent when ongoing activity (reflected in preodor fluorescence) is sufficiently high. ***B***, Same as in ***A*** but showing ITA responses of sTCs imaged from the superficial EPL in the same animal in ***A***. The field of view is directly above that shown in ***A***. The odorant is also the same as that in ***A***. Trace shows ITA and continuous recording from a suppressed sTC. This cell was suppressed by inhalation and therefore the response is the same in the presence of clean air or odorant. All white scale bars = 50 µm. Vertical dashed lines indicate inhalation onset.

We also examined inhalation-linked suppression in MTCs. Previous reports indicate that about a third of MTCs show odorant-evoked suppression of ongoing activity (Kollo et al., 2014; Economo et al., 2016; Díaz-Quesada et al., 2018). We assessed whether phasic suppression elicited by each inhalation was apparent in GCaMP6f signals, using a conservative criterion of a fluorescence decrease in the ITA of at least 3 SD below the preinhalation baseline. Using these criteria, odorant-evoked, inhalation-linked suppression was sparsely distributed and relatively rare (Fig. 4*A*), with only eight of 93 (9%) of all MC-odor pair responses and five of 240 (2%) of all sTC-odor pair responses showing suppressive ITAs. Note for these comparisons cell-odor pairs include all responses that were either significantly excited or suppressed. This prevalence is substantially smaller than the prevalence of suppressive responses seen in awake, freely-breathing mice or during higher-frequency (2 Hz) artificial inhalation (Economo et al., 2016), or as measured with whole-cell recordings (Kollo et al., 2014; Díaz-Quesada et al., 2018). A possible explanation for this difference is that the ability to detect inhalation-linked suppression using the GCaMP6f reporter was clearly dependent on baseline activity levels in individual cells, which could fluctuate over the course of a trial (Fig. 4*A*). Notably, both MCs and sTCs could also show phasic suppression linked to inhalation of clean air alone (example sTC; Fig. 4*B*). When looking across all recorded cells, suppression was more prevalent among sTCs than MCs (21 of 231 sTCs vs two of 89 MCs). Even in these cases, however, inhalation-linked suppression was sparsely distributed among the multiple cells in a field of view (Fig. 4*B*).

Overall, these data suggest that the chief difference in the inhalation-triggered dynamics of MCs versus sTCs is that MCs show a greater range of excitatory response latencies and durations than do sTCs. At the same time, they also suggest that sTC and MC responses do not unambiguously map to any single parameter of the inhalation-linked response, including response latency, response duration, or even response polarity. This finding does not contradict recent work which found that inhalation of clean air evokes distinct temporal dynamics across MC and TC populations (Fukunaga et al., 2012, 2014), as our results shown here are specific to inhalation driven dynamics in the presence of odorant. However, responses to clean air were rare in this study and thus we could not reliably compare latencies to corroborate these earlier findings. We next used this same approach to gain insight into where in the OB circuit the diversity in response patterns might arise by imaging inhalation-triggered responses from OSNs and juxtaglomerular interneurons.

### Contribution of olfactory sensory input dynamics to MTC response diversity

**Figure 5.**
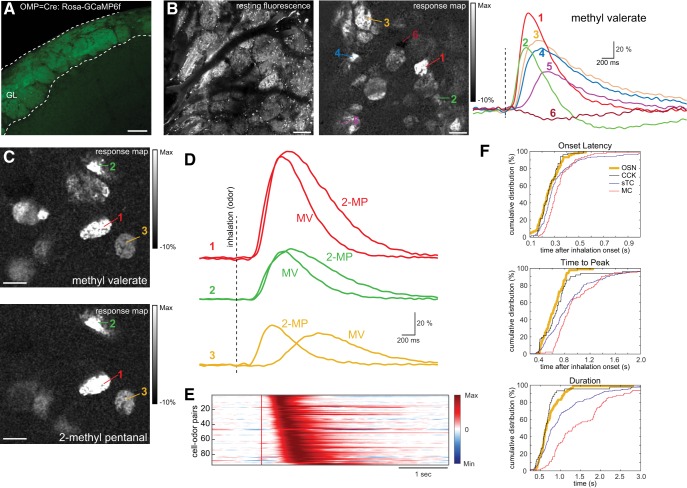
Comparison of inhalation-linked dynamics of OSN inputs and MTCs. ***A***, GCaMP6f expression in OSN axons innervating OB glomeruli as seen in confocal histology in an OMP-Cre:Rosa-GCaMP6f mouse. ***B***, *In vivo* resting fluorescence (left) and odorant-evoked ITA response map imaged across glomeruli of the dorsal OB. ITA response traces from the indicated glomeruli are shown at right. Note the range of excitatory dynamics and the presence of a suppressive response in one glomerulus. ***C***, Odor-evoked ITA response maps (left) and traces (right) showing three glomeruli that each respond to two odorants: methyl valerate (MV) and 2-methyl pentanal (2-MP). ***D***, ITA traces for each odorant are overlaid for the same glomerulus, illustrating odorant-specific response dynamics. ***E***, Pseudocolor plots of ITA responses for all odor-glomerulus pairs, displayed and normalized, as in Fig. 2, to their minimum (–1) and maximum (1) amplitude response. Red vertical line indicates inhalation onset. ***F***, Cumulative distribution plots showing OSN ITA onset latencies, times to peak, and durations, with the values for MCs, sTCs, and CCK^+^ sTCs (same data as in Fig. 2) included for comparison. All scale bars = 100 μm. Vertical dashed lines indicate inhalation onset.

One determinant of diverse MT cell inhalation-linked response dynamics could be diversity in the temporal patterns of sensory input to the OB (Spors et al., 2006). To assess this we measured the temporal dynamics of OSN input to OB glomeruli using GCaMP6f expressed in OSNs (Fig. 5*A*) and imaged responses from OSN axon terminals, as described previously (Wachowiak et al., 2013). Consistent with earlier studies (Spors et al., 2006; Carey et al., 2009; Wachowiak et al., 2013), OSN responses were predominately simple transient fluorescence increases following each inhalation (Fig. 5*B–D*). Surprisingly, inhalation of clean air elicited significant excitatory responses in only one of 72 glomeruli imaged (1/72), a lower fraction than expected given prior reports of inhalation-linked excitation among OSNs (Grosmaitre et al., 2007; Carey et al., 2009). We also observed inhalation-linked suppressive responses in a small fraction (3/72) of glomeruli imaged.

With respect to odorant-evoked activity, OSN ITAs varied in their latency of onset, rise time, and response duration, and different odorants could elicit different inhalation-triggered dynamics within the same glomerulus (Fig. 5*C*,*D*). As a population, OSN response onset latencies were distributed earlier than those of MCs, with 50% of glomerular OSN latencies preceding the shortest 82.6% of MC responses (median, 242 ms, range, 197–321 ms, *n* = 88 glomerulus-odor pairs, five mice; Fig. 5*E*,*F*). Qualitatively similar differences in temporal dynamics between OSN and MTC responses appeared for time to peak and response duration (Fig. 5*F*). The largest difference between OSN and MTC ITA dynamics was in the duration of the OSN versus MC responses, with half of all MCs showing ITA durations longer than 72.7% of all OSN responses. Notably, the distributions of onset latencies, rise times, and durations for OSN inputs overlapped closely with those of CCK^+^ sTCs (Fig. 5*F*). These results are consistent with a model in which inhalation-linked excitatory responses among sTCs, and in particular, CCK^+^ sTCs, largely reflect excitatory drive from OSNs, while MC excitation is further shaped by additional synaptic or intrinsic mechanisms (Kikuta et al., 2013; Adam et al., 2014).

### Temporal dynamics of inhalation-linked activity in juxtaglomerular interneurons

**Figure 6.**
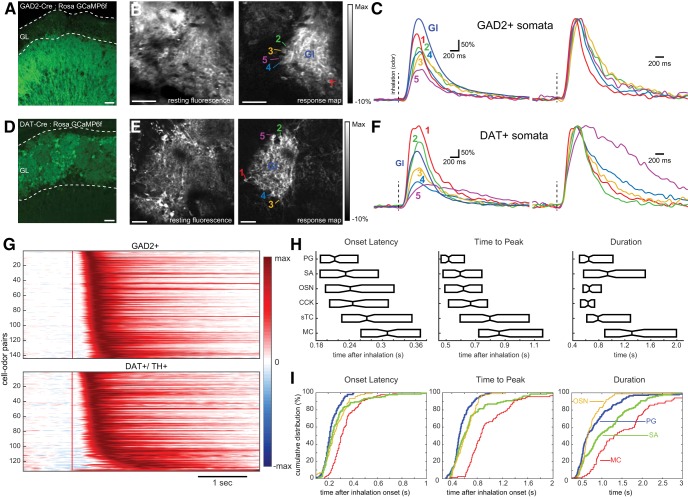
Juxtaglomerular interneurons show simple and short-latency responses to odorant inhalation. ***A***, GCaMP6f expression in the glomerular layer (GL) of a GAD2-IRES-Cre mouse, showing expression in juxtaglomerular neurons with extensive processes in the GL. Scale bar = 50 μm. ***B***, *In vivo* resting fluorescence (left) and odorant-evoked ITA response map (right) showing activation of two adjacent glomeruli in a GAD2-Cre: Rosa GCaMP6f mouse. Note that numerous somata are apparent around the periphery of the right glomerulus in the response map. Numbers indicate somata whose ITAs are shown in ***C***. Gl, glomerular neuropil. Scale bar = 50 μm. Odorant was methyl valerate. ***C***, ITA traces from the somata and glomerular neuropil from ***B***. Traces were low-pass filtered at 5 Hz before averaging. The same traces are shown scaled to the same peak values at right. Note that all cells show similar onset latencies with only modest differences in time to peak or duration. ***D–F***, Similar to ***A–C***, but for dopaminergic neurons using a DAT-Cre: Rosa GCaMP6f mouse to target presumptive SA cells. The ITA traces for these DAT+ cells are also all short-latency, with one cell showing a longer-lasting response. Odorant was 2-hexanone. Scale bars = 30 μm (***D***) and 50 μm (***E***). Vertical dashed lines indicate inhalation onset. ***G***, Pseudocolor plots of ITA responses for all cell-odor pairs for GAD2+ presumptive PG cells (top) and DAT+/TH+ presumptive SA cells (bottom), displayed and normalized as in Figure 2. Red vertical line indicates inhalation onset. ***H***, Box plot comparisons of ITA onset latencies, time to peak, and half width durations. Medians = center horizontal line. Boxes represent 2nd and 3rd quartiles of each cell populations. ***I***, Cumulative distribution plots of onset latencies, times to peak, and durations for GAD2+ and DAT/TH+ cells highlighted by thicker plots. OSN and MTC populations shown for reference.

We next characterized inhalation-linked responses in juxtaglomerular interneurons, focusing on PG and SA cells, these two classes of interneurons are hypothesized to shape MTC responses via feedforward and lateral inhibition (Kosaka and Kosaka, 2008; Liu et al., 2013, 2016a; Fukunaga et al., 2014; Banerjee et al., 2015). GCaMP6f was preferentially targeted to PG or SA cells by either virus injection (AAV.Flex.GCaMP6f) or Rosa-GCaMP6f reporter cross using GAD2-Cre mice (for PG cells) or TH-Cre or DAT-Cre mice (for SA cells), as described previously (Wachowiak et al., 2013; Banerjee et al., 2015; Fig. 6*A*,*D*). Calcium signals were imaged from somata located around the glomerulus periphery (Fig. 6*B*,*E*).

Both GAD2+ PG and TH/DAT+ SA cell ITA odor responses consisted overwhelmingly of simple, monophasic response transients; multiphasic responses were not seen (Fig. 5*C*,*F*). In contrast to MCs and sTCs, odorants did not elicit suppressive responses in 1/145 PG cell-odor pairs or 0/132 SA cell-odor pairs. When examining individual cells, clean air did elicit excitatory responses in a small fraction of both cell types (7/136 PG cells; 2/120 SA cells). With respect to odorant-evoked response dynamics, PG and SA cell populations both had short-latency ITAs, with the main difference being a “tail” of longer-latency SA cell responses (Fig. 5*I*,*J*). Indeed, the median PG cell ITA latency preceded that of 94% of all MCs, whereas the median SA cell ITA latency preceded 88% of all MCs. At the population level, OSN inputs, PG, SA cells, and CCK^+^ sTC all had ITA onset latencies that were statistically similar to each other (Kruskal–Wallis test, χ^2^ = 100.45, df = 715, *p*>χ^2^ = 4.2e−20, Tukey–Kramer *post hoc* test, *p* > 0.05) and significantly faster than that of MCs and other sTCs (Tukey–Kramer *post hoc* test, *p* < 0.05; Fig. 5*I*,*J*, left panels). Similar trends were observed when comparing time to peak response across these populations (Kruskal–Wallis test, χ^2^ = 162.05, df = 715, *p*>χ^2^ = 3.6e−33; Fig. 5*I*,*J*, middle panels).

SA cell responses differed from those of PG cells mainly in their response durations (PG half width median, 646 ms, interquartile range: 504–1018 ms, SA median, 935 ms, interquartile range: 566–1514 ms; Fig. 5*I*,*J*, right panels), with SA cell durations shifted toward longer values than those of PG cells (Fig. 5*I*,*J*, right panels). Indeed, there was no significant difference in response duration of OSNs, PG cells, and CCK^+^ sTCs (Kruskal–Wallis test, χ^2^ = 104.62, df = 698, *p*>χ^2^ = 5.6e−21, Tukey–Kramer *post hoc*, *p* > 0.05), while SA cell response durations were significantly longer than all three of these populations (Tukey–Kramer *post hoc*, *p* < 0.05). Interestingly, SA and sTC response durations were statistically indistinguishable (Tukey–Kramer *post hoc*, *p* > 0.05). The similar inhalation-linked dynamics among these groups, which encompasses axon terminals, small-sized PG somata and larger-sized sTC somata suggests that variation in calcium dynamics, as a function of cellular compartment or soma size is not a major determinant of the response dynamics seen after odorant inhalation. Overall, these results suggest that inhalation-linked PG and SA responses largely follow those of OSN inputs, while a subset of SA cells exhibit longer-lasting responses. Their short onset latencies are consistent with both cell types mediating rapid feedforward inhibition of MCs and sTCs during inhalation.

### The diversity of inhalation-linked response dynamics within a single glomerulus

PG cells, sTCs, and MCs all receive excitatory input from dendrites confined to a single glomerulus, and there is evidence that different MCs associated with the same glomerulus (i.e., sister MCs) can show distinct temporal response patterns (Dhawale et al., 2010; Arneodo et al., 2018), while sister PG cells have been reported to show temporally uniform response latencies (Homma et al., 2019). We thus next asked to what degree does variance in inhalation-linked response patterns reflect heterogeneity among sister PG cells or sTCs, as opposed to simply reflecting glomerulus-specific and odorant-specific diversity in OSN input dynamics (Spors et al., 2006).

**Figure 7.**
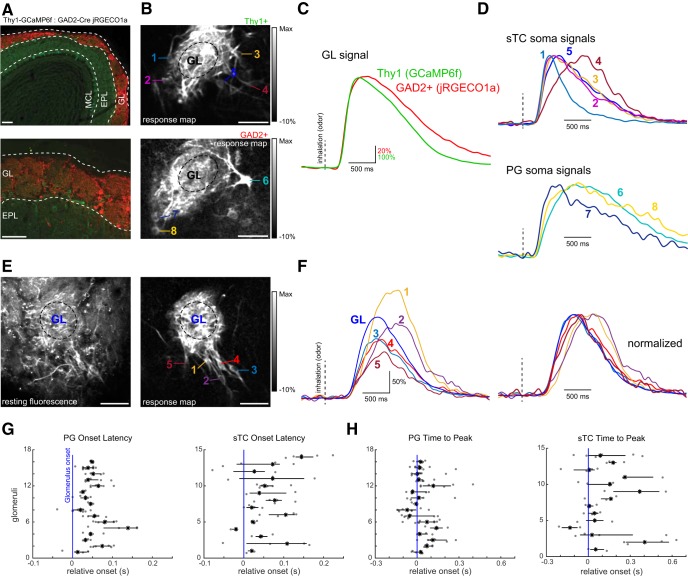
Diversity of inhalation-linked temporal dynamics is greater in sTC than PG cells innervating the same glomerulus. ***A***, Tissue sections showing GCaMP6f expression (green) in Thy1+ MTCs (green) and jRGECO1a expression in GAD2+ juxtaglomerular neurons, after Flex.jRGECO1a (red) virus injection in a Thy1-GCaMP6f:GAD2-Cre mouse (see Text). Scale bar = 100 μm. ***B***, Odorant-evoked ITA response map showing activation of MTCs recorded in the green channel (top) and GAD2+ neurons recorded simultaneously in the red channel (bottom). Numbers indicate presumed sTCs or PG cells whose responses are shown in ***D***. Odorant was ethyl butyrate. Scale bar = 50 μm. ***C***, Overlay of ITA traces for the Thy1+ and GAD2+ signals recorded from the neuropil of the glomerulus shown in ***B***. Traces normalized to their maximum response to highlight temporal differences. ***D***, ITA traces from the somata indicated in ***B***, with presumptive sTCs (top) and PG cells (bottom) overlaid with each other. All cells appear to innervate the same glomerulus. A 5-Hz low-pass filter was applied to all traces before averaging. Traces are normalized to their maximum response. ***E***, Resting fluorescence (left) and odorant-evoked ITA response map (right) taken from a Tbet-Cre mouse expressing GCaMP6f, showing a group of sister sTCs innervating the same glomerulus. Odorant was methyl valerate. Scale bar = 50 μm. ***F***, ITA response traces from the sTCs shown in ***E***, along with the glomerular signal (GL). Traces shown at right are normalized to their peak response to illustrate diversity in inhalation-linked rise times, and durations. Vertical dashed lines indicate inhalation onset. ***G***, Dot plots showing the distribution of onset latencies for sister PG cells (left) or sTCs (right) associated with the same glomerulus, referenced to the latency measured from the neuropil of the parent glomerulus (blue vertical line). Each row is a different glomerulus. Black horizontal line spans the interquartile range and the black asterisk is the median. ***H***, Same analysis in ***G*** but for time to peak for the same cells and glomeruli.

Initially, we directly compared PG and sTC response dynamics during the same odor stimulus and within the same glomerulus using two-color imaging. We used Thy1-GCaMP6f: GAD2-Cre crosses, expressing the red shifted calcium indicator jRGECO1a (Dana et al., 2016) in PG cells using a Cre-dependent viral vector (Fig. 7*A*). Separate excitation lasers and selective emission filters (see Methods) were used to simultaneously and selectively image from Thy1+ sTCs and GAD2+ PG cells in the same field of view or from the neuropil of the same glomerulus (Fig. 7*B*). Signals imaged from the glomerular neuropil were generally similar for the Thy1+ signal, which reflected summed activity in mitral as well as tufted cell primary dendrites, and the GAD2+ signal, which reflected summed activity across PG cell processes (23 glomeruli, three mice; Fig. 7*C*). The slower decay in the GAD2+ signal is consistent with the slower decay of jRGECO1a after a calcium transient as compared to GCaMP6f (Dana et al., 2016). However, imaging from individual PG and sTC somata associated with the same glomerulus revealed diversity in inhalation-triggered dynamics, with different cells showing distinct time to peak and response durations (Fig. 7*D*). While the numbers of sister PG and sTC cells imaged from the same glomerulus were not sufficient for strong statistical analysis, these observations directly demonstrate that distinct inhalation-triggered temporal dynamics can emerge among different neurons innervating the same glomerulus.

To more systematically compare this diversity of PG cells and sTCs, we returned to single-wavelength imaging using GCaMP6f, focusing on collecting data from sister PG (Fig. 6*B*,*C*) or sTC somata (Fig. 7*E*,*F*) in separate preparations. We assessed heterogeneity among sister cells of each cell type by computing the SD (σ) of the ITA onset latencies and times to peak across all cells associated with a single “parent” glomerulus (Fig. 7*G*,*H*). Onset latencies and times to peak were more variable across sister sTCs than for PG cells: the σ for sTC and PG cell of sister sTC onset latencies was 45 ± 9 ms (mean ± SEM, *n* = 14 glomeruli from six mice) compared with 19 ± 0.01 ms (*n* = 16 glomeruli from four mice) for PG cells (*p* = 0.008, unpaired *t* test; Fig. 7*G*), consistent with a recent report (Homma et al., 2019); the SD of times to peak (same glomeruli and cells as above) was 123 ± 20 ms for sTCs, compared with 73 ± 36 ms for PG cells (*p* = 0.025, unpaired *t* test; Fig. 7*H*). As expected, the variance among sister PG or sTCs was less than the variance across the entire population of imaged neurons (σ for sTC and PG cell onset latencies, 217 and 61 ms, respectively; σ for sTC and PG cell times to peak, 430 and 140 ms), but was still substantial. These results suggest that the greater variability in inhalation-triggered response dynamics observed across the population of sTCs as compared to PG cells is not merely an artifact of sampling across different glomeruli, but instead that this diversity can emerge within the glomerular circuit.

## Discussion

A single inhalation of odorant is sufficient for odor identification, and incoming olfactory information arrives at the OB in the form of transient bursts of OSN activity linked to each inhalation. The neural circuits that process olfactory inputs are well known, but how these circuits respond to the dynamic inputs driven by odorant inhalation *in vivo* remains unclear. Here, we sought to better understand this key processing step by imaging from major cell types in the olfactory network and sampling odorants in the anesthetized mouse using a standard, reproducible inhalation. This approach allowed us to compare the dynamics of inhalation-linked activity as it progressed through the OB glomerular network, beginning with OSN inputs and glomerular layer interneurons thought to perform key sensory processing early in the respiratory cycle, and ending with MTCs, which carry information out of the OB.

Several general principles emerged. First, inhalation elicits relatively simple bursts of OSN input to a glomerulus, which occur over a limited range of latencies that is glomerulus and odor specific. Second, juxtaglomerular inhibitory interneurons, e.g., presumptive PG and SA cells, also show uniformly short onset latencies and simple excitatory response transients following inhalation. Third, diversity in inhalation-linked response patterns emerges at the level of glomerular output neurons, manifesting in a larger range of times to peak response and burst durations and in a higher prevalence of suppressive components of the inhalation-linked response. Finally, we find that MTC response patterns are highly overlapping, such that these projection neuron subtypes cannot be cleanly distinguished solely on the basis of their inhalation-linked responses. Overall, these results are consistent with a model in which diversity in inhalation-linked patterning of OB output arises first at the level of OSN inputs to the OB and is then enhanced by feedforward inhibitory circuits in the glomerular layer (Dhawale et al., 2010; Kikuta et al., 2013).

The glomerulus-specific and odorant-specific variation in inhalation-linked response latencies of OSN inputs is consistent with that described earlier by us and others (Spors et al., 2006; Carey et al., 2009), with latencies varying across a range of 197–321 ms (25th–75th percentiles). Notably, PG and SA cells showed a near-identical distribution of response patterns, with responses overwhelmingly consisting of simple and brief inhalation-driven bursts of excitation. However, a fraction of SA cells displayed response durations that were prolonged relative to those of PG cells. In contrast, diversity in inhalation-locked MTC activity could not be fully accounted for by diversity in OSN inputs. Both MTC populations displayed more delayed inhalation-linked onset latencies and larger range of time to peak and burst durations than seen among OSNs, and inhalation-linked response patterns could include multiphasic excitatory components, features which were rare or absent among OSNs.

Our data also allowed us to compare inhalation-linked response patterns of mitral versus tufted cells. Surprisingly, at the population level, we found little difference in inhalation-linked dynamics between these two populations. We did observe that the prevalence of clean air evoked ITA responses was greater among sTCs. Furthermore, sTCs defined by their expression of the neuropeptide transmitter CCK showed a significantly shorter range of response latencies and durations than mitral cells or the wider population of sTCs. However, the distribution of sTC and mitral cell response patterns overlapped a great deal: mitral and sTCs (as defined by soma location regardless of genetic marker) were not different in mean onset latencies, and the mode of their latency distribution was identical for the two cell types. Some earlier studies have reported clear differences in the latencies of mitral versus tufted cell responses to inhalation of air, although these timing differences disappear during odorant stimulation (Fukunaga et al., 2012, 2014); thus our results are not inconsistent with these recent reports. Finally, while relatively rare compared to earlier reports (Kollo et al., 2014; Economo et al., 2016; Díaz-Quesada et al., 2018), inhalation-linked suppression was seen in odorant responses of both mitral cells and sTCs. Overall, our data are consistent with the long-held notion that MTCs constitute functionally distinct subpopulations of output neurons, but indicate that these cell types cannot be distinguished solely on the basis of their inhalation-linked responses. Instead, our data suggest that the representation of olfactory information by these subpopulations, at least with respect to the temporal dynamics of odor-evoked activity following inhalation, is highly overlapping.

Respiratory patterning of ongoing activity in the absence of odorant stimulation is well-documented and has been hypothesized, among other functions, to serve as a reference for a timing-based code for odor identity (Kepecs et al., 2006; Spors et al., 2006; Cury and Uchida, 2010; Shusterman et al., 2011; Wachowiak, 2011). Here, we observed inhalation-linked patterning of activity during inhalation of clean air in all OB cell types, although the prevalence of such responses (<10% across all populations) was smaller than reported in earlier studies (Fukunaga et al., 2014; Díaz-Quesada et al., 2018). Indeed, in this study, excitation in response to inhalation was unique to sTCs and not observed among MCs. This lower prevalence may be a result of our use of cleaned air rather than ambient room air as our background condition, or may reflect limitations in the sensitivity and temporal resolution of the GCaMP imaging approach.

Surprisingly, a small fraction of clean air-driven responses were suppressive, with such suppression observed in sTCs, MCs, and even some OSN inputs. To our knowledge, this is the first report of inhalation alone driving suppression of activity in these cell types. One explanation for these results could lie in the low frequency (0.25 Hz) of artificial inhalation used in our experiments: if some OSN populations are sensitive to odor components arising from within the animal’s own nasal cavity, e.g., from metabolic processes, these components could drive basal activity of OSNs and in sTCs of their target glomeruli, which would be transiently removed by each inhalation of clean air. This effect could be even more pronounced in the intact, awake mouse where OSNs are exposed to exhaled air containing metabolic odorants (Munger et al., 2010; Mori et al., 2014).

What can we infer from these comparisons about the primary synaptic interactions shaping inhalation-linked patterning of OB output? First, the data suggest that PG and SA cell excitation largely follows OSN input dynamics, consistent with evidence from slice studies that these cells are highly sensitive to OSN stimulation mediated either by monosynaptic or disynaptic excitation (Gire and Schoppa, 2009; Shao et al., 2009; Kiyokage et al., 2010; Najac et al., 2015). We saw little to no evidence of delayed PG/SA cell responses that would correspond to feedback excitation from the late-phase responses observed in some MTCs. In fact, only 2.3% of PG and 7.4% of SA interneuron onset latencies followed the mean M/sTC onset latency. This result is somewhat surprising, as mitral cells can mediate feedback excitation of PG and SA cells via dendrodendritic synapses in the glomerular neuropil (Najac et al., 2015). Second, our results suggest that rapid feedforward inhibition from PG or SA cells may underlie the longer-latency responses seen in some MTCs, e.g., the slowest quartile of MT responses show onset latencies that roughly match the median peak time of the PG cell response. Third, the presence of longer-duration mitral cell excitatory responses that outlast those of any OSN input suggests an additional source of excitatory drive onto mitral cells, the identity of which remains unclear. A disinhibitory circuit is unlikely, as we did not observe suppression of PG or SA cells; therefore, these results suggest that the intrinsic properties of MT cells could give rise to prolonged spike bursts, or that secondary excitation by ET cells could extend the duration of M/sTC excitatory responses (Carlson et al., 2000). This prolonged excitatory component was often longer than the 4-s interval between sniffs, implying that it may be important in shaping tonic levels of excitability across multiple inhalations in the awake animal (Díaz-Quesada et al., 2018).

Overall, these results establish a basic framework for how glomerular circuits are engaged to shape inhalation-linked patterning of OB output. Our findings support the hypothesis that feedforward inhibitory circuits can add to the initial diversity of temporal patterns of input relayed by OSNs. additional experiments are necessary to integrate other OB cell types into this framework, e.g., granule cells, deep SA cells, external plexiform layer interneurons and centrifugal inputs from the olfactory cortex may also contribute to shaping respiratory patterning of OB output. Understanding the response dynamics of each of these cell types with respect to a single inhalation of odorant should allow for a dynamic model of OB network function across the fundamental unit of information sampling in the olfactory system. Such a model may be used to yield insights into olfactory processing across the full range of sampling frequencies used in the behaving animal.

## Synthesis

Reviewing Editor: Anna Menini, SISSA (International School of Advances Studies) Decisions are customarily a result of the Reviewing Editor and the peer reviewers coming together and discussing their recommendations until a consensus is reached. When revisions are invited, a fact-based synthesis statement explaining their decision and outlining what is needed to prepare a revision will be listed below. The following reviewer(s) agreed to reveal their identity: NONE. Note: If this manuscript was transferred from JNeurosci and a decision was made to accept the manuscript without peer review, a brief statement to this effect will instead be what is listed below.

The authors answered previous reviewers' comments and made the suggested revisions.
